# Does particle radiation have superior radiobiological advantages for prostate cancer cells? A systematic review of in vitro studies

**DOI:** 10.1186/s40001-022-00942-2

**Published:** 2022-12-26

**Authors:** Tian-Qi Du, Ruifeng Liu, Qiuning Zhang, Hongtao Luo, Yanliang Chen, Mingyu Tan, Qian Wang, Xun Wu, Zhiqiang Liu, Shilong Sun, Kehu Yang, Jinhui Tian, Xiaohu Wang

**Affiliations:** 1 Institute of Modern Physics, Chinese Academy of Sciences, 509 Nanchang Rd, Lanzhou, 730000 Gansu People’s Republic of China; 2 The First School of Clinical Medicine, Lanzhou University, Lanzhou, Gansu People’s Republic of China; 3 Graduate School, University of Chinese Academy of Sciences, Beijing, People’s Republic of China; 4 Heavy Ion Therapy Center, Lanzhou Heavy Ion Hospital, Lanzhou, Gansu People’s Republic of China; 5 Evidence-Based Medicine Center, School of Basic Medical Sciences, Lanzhou University, Lanzhou, Gansu People’s Republic of China

**Keywords:** Prostate cancer, In vitro, Charged particle irradiation, Systematic review

## Abstract

**Background:**

Charged particle beams from protons to carbon ions provide many significant physical benefits in radiation therapy. However, preclinical studies of charged particle therapy for prostate cancer are extremely limited. The aim of this study was to comprehensively investigate the biological effects of charged particles on prostate cancer from the perspective of in vitro studies.

**Methods:**

We conducted a systematic review by searching EMBASE (OVID), Medline (OVID), and Web of Science databases to identify the publications assessing the radiobiological effects of charged particle irradiation on prostate cancer cells. The data of relative biological effectiveness (RBE), surviving fraction (SF), standard enhancement ratio (SER) and oxygen enhancement ratio (OER) were extracted.

**Results:**

We found 12 studies met the eligible criteria. The relative biological effectiveness values of proton and carbon ion irradiation ranged from 0.94 to 1.52, and 1.67 to 3.7, respectively. Surviving fraction of 2 Gy were 0.17 ± 0.12, 0.55 ± 0.20 and 0.53 ± 0.16 in carbon ion, proton, and photon irradiation, respectively. PNKP inhibitor and gold nanoparticles were favorable sensitizing agents, while it was presented poorer performance in GANT61. The oxygen enhancement ratio values of photon and carbon ion irradiation were 2.32 ± 0.04, and 1.77 ± 0.13, respectively. Charged particle irradiation induced more G0-/G1- or G2-/M-phase arrest, more expression of γ-H2AX, more apoptosis, and lower motility and/or migration ability than photon irradiation.

**Conclusions:**

Both carbon ion and proton irradiation have advantages over photon irradiation in radiobiological effects on prostate cancer cell lines. Carbon ion irradiation seems to have further advantages over proton irradiation.

**Graphical Abstract:**

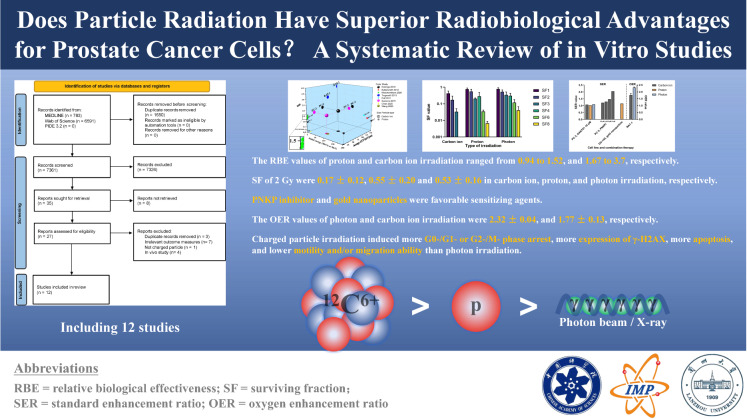

**Supplementary Information:**

The online version contains supplementary material available at 10.1186/s40001-022-00942-2.

## Introduction

Globally, there are estimated to be 1,414,259 new cases and 375,304 deaths from prostate cancer (PCa) in 2020, which is the second most frequent malignancy in males and the fifth leading cause of cancer mortality among men worldwide [1]. Radiation therapy (RT) has been widely used to treat PCa for many years. Given that PCa control is dose-dependent, intensity-modulated RT (IMRT), image-guided RT and brachytherapy can result in an adequate dose to the prostate or prostate bed while causing less gastrointestinal and genitourinary toxicity, especially in the rectum and bladder [2–4]. Charged particle therapy (CPT) for PCa gained increasing attention in the past decade because of its superior physical and biological properties. Several studies have shown a reduced radiation toxicity, lower risk of secondary primary tumor and an improved biochemical-free survival (also known as biochemical disease-free survival) compared with IMRT [5–14]. In these studies, despite the lack of high-quality randomized controlled trials, existing data suggested that CPT exhibited great potential for sparing normal tissue as well as excellent overall survival rate and local control rate [15]. CPT may become a promising approach to treat PCa someday in the years to come.

The physical properties of carbon ions and protons are quite similar. With the presence of depth-dose distribution, also known as the Bragg peak, CPT can give the maximum energy to the surroundings near the stop (cancer part) [16]. In addition to accommodate the tumor volume, a spread-out Bragg peak (SOBP) is also produced. Carbon ions exhibit less lateral scattering and longitudinal straggling than protons, but they have a tail of light fragments beyond the Bragg peak, and nuclear fragmentation causes a drop in dose in the plateau area [17]. As for the biological properties, due to the higher linear energy transfer (LET) and relative biological effectiveness (RBE) of carbon ions compared with protons and photons, they can directly cause complex damage to DNA molecules and cause clustered DNA double-strand breaks (DSBs), requiring multiple DNA repair pathways to resolve [18]. Additionally, as a result of this damage, the oxygen enhancement ratio (OER) of carbon ions is typically ranged between 1 and 2.5, depending on the LET value, whereas the OER of photons and protons is generally estimated to be as high as 3 [19].

Due to these particular features of charged particles, some in vitro studies have focused on the radiobiological effects of carbon ion and proton irradiation on PCa. However, most of these studies have different relevant parameters, such as charged particle type, cell line type, cell origin, energy, LET, SOBP, combination therapy and so forth. Therefore, we believe it is necessary to pool these studies for further analysis. In this paper, we conducted a systematic review based on published in vitro studies of carbon ion or proton irradiation for PCa to study its biological mechanisms regarding both traditional radiobiology and molecular level.

## Materials and methods

This systematic review was performed in accordance with the guidelines proposed by the Preferred Reporting Items for Systematic Reviews and Meta-Analyses Additional file 3 (PRISMA) 2020 statement [20]. As our review contained only in vitro studies and did not involve any human or animal studies, it did not meet the criteria for registration on PROSPERO website and therefore we were unable to register in.

### Search strategy

The following electronic databases were used as our data sources: MEDLINE (Ovid MEDLINE(R) and Epub Ahead of Print, In-Process, In-Data-Review and Other Non-Indexed Citations, Daily and Versions(R) < 1946 to February 18, 2022 >), Embase (< 1974 to 2022 February 18 > , Ovid) and Web of Science databases (WOS, BIOSIS, KJD, RSCI, SCIELO). The following search terms were used: “heavy ion radiotherapy”, “heavy ion therapy”, “heavy ion radiation therapy”, “particle beam therapy”, “carbon ion therapy”, “carbon ion radiation therapy”, “carbon ion radiotherapy”, “carbon ion irradiation”, “proton therapy”, “proton radiation”, “proton irradiation”, “prostatic neoplasms”, “prostate cancer”, “prostatic cancer”, “prostate adenocarcinoma” and “prostatic adenocarcinoma”. Specific search strategies for each database were listed in Additional file 1. Furthermore, we searched Particle Irradiation Data Ensemble Version 3.2 (PIDE 3.2), a database including in vitro data for ions and cell lines established by GSI (https://www.gsi.de/bio-pide). We also screened all the references in the included studies to ensure no available publications were omitted.

### Literature selection and criteria

We used the following selection criteria: (1) articles reporting in vitro studies of PCa cell lines irradiated by carbon ion or proton beam; (2) articles reporting at least one of these following outcomes: (i) cell clonogenic survival; (ii) DNA damage response and repair (DDR/R) (e.g., cell cycle checkpoints, DSB repair, or apoptosis); (iii) motility, migration or invasion; (iv) OER or standard enhancement ratio (SER) evaluating the effect of combination therapy on colony forming assay; (3) articles published in English. Articles not matched the selection criteria were excluded. Other exclusion criteria included the following: (1) using artificially modified cells lines; (2) review, editorial material, comment or conference/meeting abstract; (3) pilot studies and research projects; and (4) full text was not available.

After we imported the retrieved articles (as several RIS format files from different sources) into EndNoteX9 software, the duplicate publications were excluded automatically. Two trained investigators independently did the literature screening by reading the titles and abstracts, then evaluated potential full texts and determined eligibility. All conflicts were resolved by discussion with a senior investigator to achieve consensus.

### Data extraction

After pilot testing our predefined data extraction forms, two trained investigators independently extracted relevant data from the studies. The main contents of them included general characteristics (first author, year of publication and country), irradiation information (particle type, particle accelerator facility or institution, initial energy, average LET, SOBP, dose rate, and dose group), cell line type and origin (human or animal), RBE, survival fraction (SF), SER of different combinations therapy and OER. All conflicts were resolved by discussion with a senior investigator to achieve consensus.

When the article failed to specify SF, then data were extracted from the survival curves in published plots using Web Plot Digitizer Version 4.5 (https://automeris.io/WebPlotDigitizer) to convert datapoints into numerical values, or calculated by following formula:

$$SF={e}^{-(\alpha D+\beta {D}^{2})}$$

where *D* is the delivered dose, *α* and *β* are fitting constants representing the initial slope and the curvature of the survival curve if they were reported. When the article failed to specify SER, data were extracted in the same way, then determined the SER by calculating the ratio of doses at 10% survival level (SF = 0.1) in treated and control groups (namely, SER_10_, but abbreviated as SER in this article) [21].

### Risk of bias assessment

As of today, there is still no accepted standard risk of bias assessment tool to refer to for in vitro studies. Our team has established these criteria by ourselves after referring to some acknowledged risk-of-bias tools [22–24]. In this review, we refined and updated the content over the last version (Additional file 2: Table S1). Two trained investigators independently assessed the risk of bias, and all conflicts were resolved by discussing with a senior investigator to achieve consensus.

### Statistical analysis

All the parameters we extracted were conducted as descriptive statistics. The continuous data from RBE, SF, SER and OER were represented as the mean ± standard deviation or median with interquartile ranges. All analyses were done using GraphPad Prism software (version 9.3.0) and R statistical software (version 4.1.2).

## Results

### Search results

**Fig. 1:**
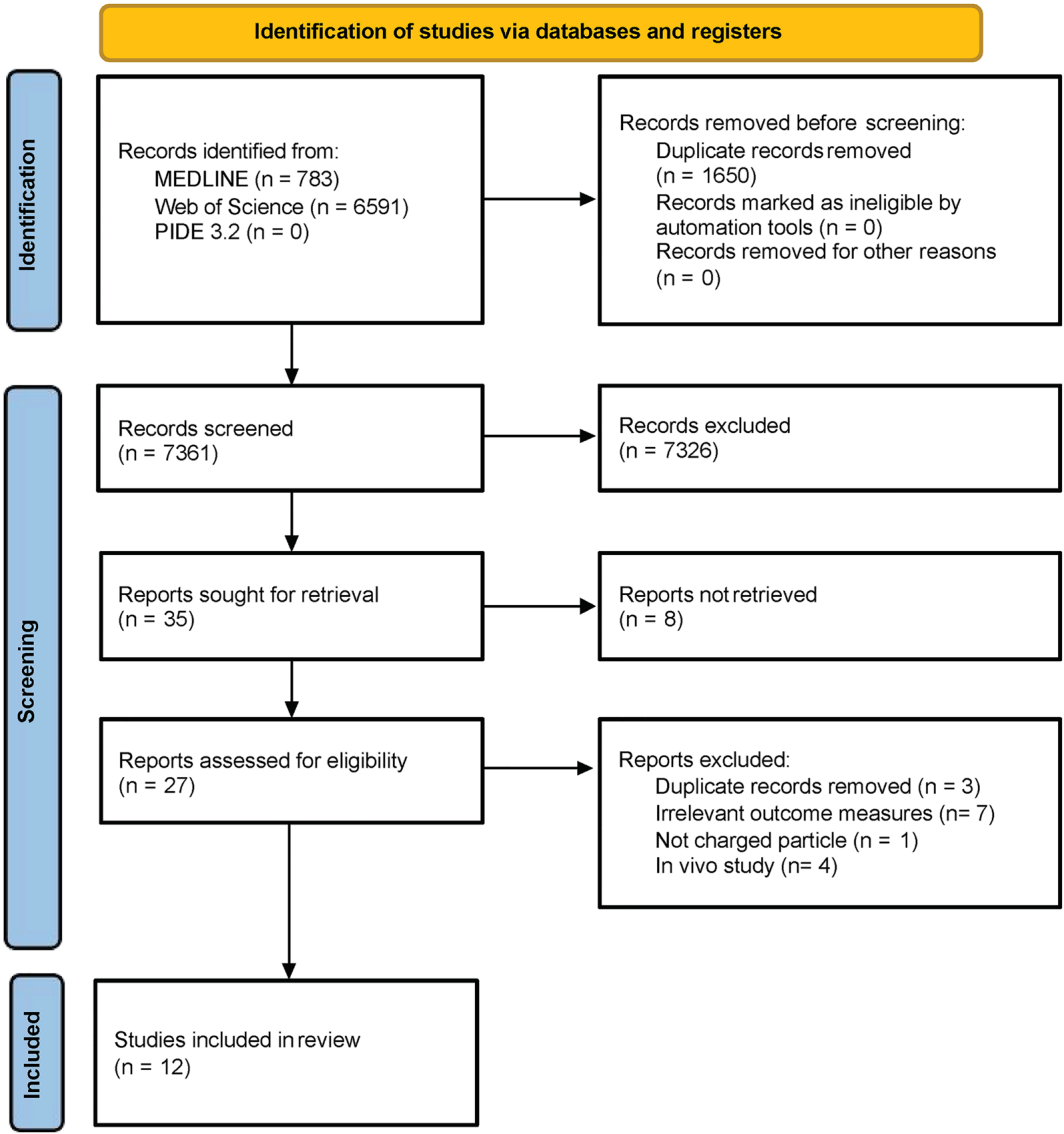
PRISMA flow diagram of the systematic review. *PRISMA* Preferred Reporting Items for Systematic Reviews and Meta-Analyses

Our systematic search identified 7361 records were potentially eligible for review after 1650 duplication records were removed. 7326 records were excluded based on the screening of the titles and abstracts. Of the remaining 35 records, 8 failed to retrieve the full text because all of them were conference or meeting abstracts. Full texts of 27 records were read, ultimately, a total of 12 studies (7 for carbon ion irradiation, 4 for proton irradiation and 1 for both) met the inclusion criteria (Fig. 1).

### Study characteristics

The included studies were published from 2011 to 2020, seven were on carbon ion irradiation [25–31], four on proton irradiation [32–35] and one on both [36]. These studies were completed in seven countries: four of them in Belgium [26–28, 36], three in China [30, 31, 33], and one each in India [25], the United Kingdom [32], Germany [29], Austria [34], and the United States of America [35]. Almost all of the cell lines used in these studies were of human origin: PC-3 [25–28, 31, 33, 36], DU-145 [32, 34, 35], LNCap [30], only one used rat origin RAT-1 cell line [29]. The studies varied in the initial energy, averaged LET, SOBP, dose groups, and dose rate used. It should be noted that only 4 studies reported the SOBP value [29, 32, 35, 36], and 1 used monoenergy [33], 1 used multi-energy but no SOBP value (carbon ion beam) [36], the other studies unspecified monoenergy or multi-energy. 3 studies combined with drugs, such as GLI antagonist (GANT61, inhibitor of GLI1/2), polynucleotide kinase/phosphatase inhibitor (PNKPi) and gold (Au) nanoparticles (GNPs) [25, 35, 36]. One study demonstrated the effect of acute oxygen depletion on cell survival for different types of radiation [29]. At last, 9 control groups were treated using X-ray [26–29, 31, 32, 34–36], and the other 3 control groups were not irradiated. The basic characteristics of the included studies are summarized in Table 1.

**Table 1:** Overview of the different experimental parameters used in the studies

Author (year)	Country	Charged particle	Accelerator facility or institution; location	Cell type	Cell origin	Initial energy	Average LET (keV/µm)	SOBP	Dose rate	Dose group	Combination therapy	Control
Wang 2019	China	Carbon Ion	HIRFL; Lanzhou, China	LNCaP	Human	80 ~ 100 MeV/u	NA	NA*	1 Gy/min	0.5, 4 Gy	NA	No IR
Konings 2019	Belgium	Carbon Ion And Proton	GANIL; Caen, France iThemba LABS; Cape Town, South Africa	PC-3	Human	95 MeV/u 200 MeV	73 3.96	NA† 50 mm	NA	0, 0.25, 0.5, 1, 2, 3, 4 Gy 0.25, 0.5, 2, 4, 6 Gy	GANT61	X ray
Srivastava 2018	India	Carbon Ion	IUAC; New Delhi, India	PC-3	Human	7.08 MeV/u	NA	NA*	NA	0, 2, 4 Gy	PNKPi	No IR
Butterworth 2012	UK	Proton	MGH; Boston, USA	DU-145	Human	178 MeV	NA	45 mm	NA	0.5, 1, 2, 4, 6 Gy	NA	X ray
Khachonkham 2020	Austria	Proton	NA	DU-145	Human	127.2–180.1 MeV	1.9, 2.5, 4.1 (within SOBP); 1.0, 1.2 (before peak)	NA*	NA	0, 0.5, 1, 2, 4, 6 Gy	NA	X ray
Tinganelli 2013	Germany	Carbon Ion	GSI; Darmstadt, Germany	RAT-1	Rodent	NA	100, 150	10 mm	NA	NA	NA	X ray
Suetens 2016	Belgium	Carbon Ion	GANIL; Caen, France	PC-3	Human	75 MeV/u	33.7	NA*	NA	0, 0.5, 1, 2 Gy	NA	X ray
Polf 2011	USA	Proton	NA	DU-145	Human	160 MeV	NA	100 mm	NA	0, 1, 2, 3, 4, 6 Gy	Gold nanoparticles	X ray
Suetens 2015	Belgium	Carbon Ion	GANIL; Caen, France	PC-3	Human	75 MeV/u	33.7	NA*	NA	0, 0.5, 2.0 Gy	NA	X ray
Chen 2020	China	Proton (Microbeam)	NIRS; Chiba, Japan	PC-3	Human	3.4 MeV	11.7	monoenergy	NA	0, 100, 250, 500 protons	NA	No IR
Wang 2020	China	Carbon Ion	HIRFL; Lanzhou, China	PC-3	Human	81 MeV/u	32.54	NA*	2 Gy/min	0, 1, 2, 4, 6, 8 Gy	NA	X ray
Suetens 2014	Belgium	Carbon Ion	GANIL; Caen, France	PC-3	Human	75 MeV/u	33.7	NA*	NA	0, 0.5, 2.0 Gy	NA	X ray

*LET* linear energy transfer, *SOBP* spread-out Bragg peak, *IR* irradiation, *GANT61* GLI antagonist, *PNKPi* polynucleotide kinase/phosphatase inhibitor, *NA* not available

^a^ Unspecifying monoenergy or multi-energy

^b^ Multi-energy but without SOBP value

### Risk of bias

**Fig. 2:**
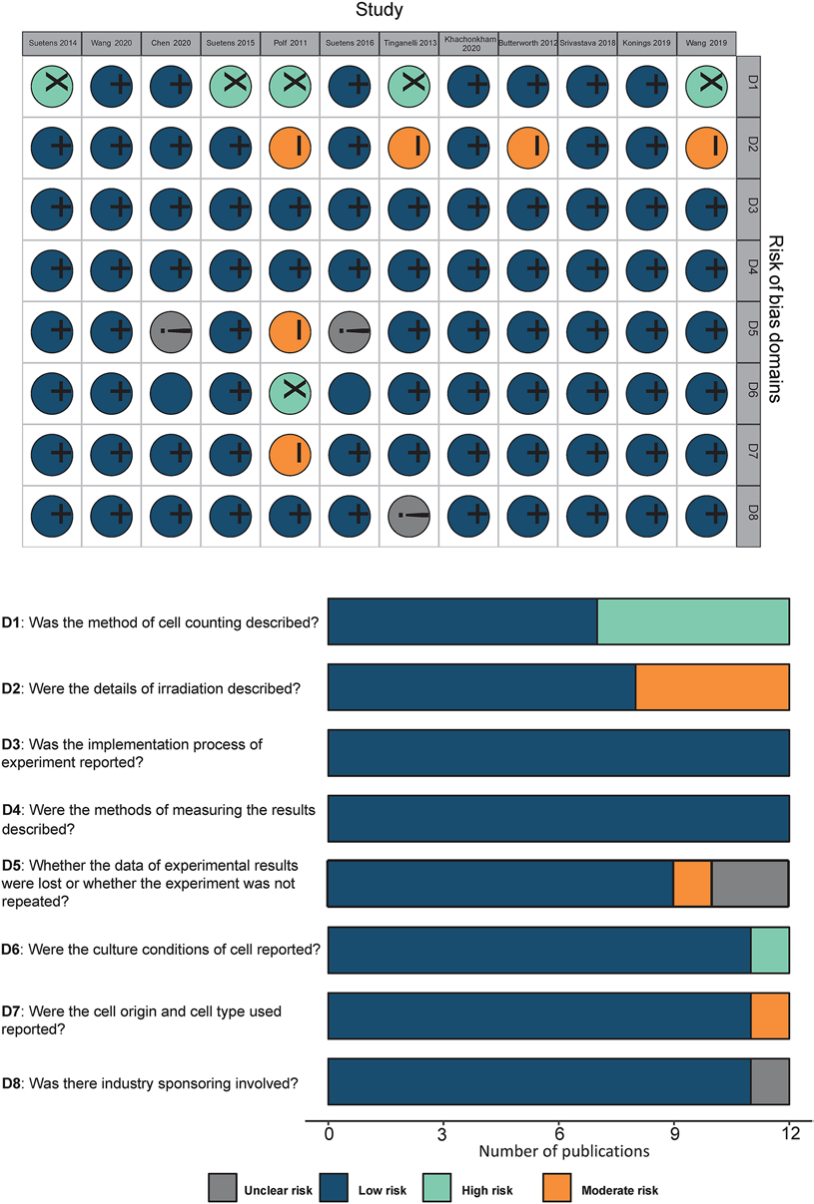
Results of the risk of bias assessment

There were five studies did not describe the method of cell counting, resulting in high risk of selection bias [27–30, 35]. All 12 studies reported the implementation process of experiment; but four studies did not describe the details of irradiation completely, resulting in moderate risk of performance bias [29, 30, 32, 35]. All 12 studies described the methods of measuring the results. 1 study did not repeat the data of experiment results, resulting in moderate risk of attrition bias [35], and 2 studies did not report whether the experiments were repeated [26, 33]. Only 1 study did not report the culture conditions of the cell and cell origin, resulting in a high and a moderate risk of cell-related bias [35]. In addition, one study failed to specify whether there was industry sponsoring involved [29]. These mentioned above results of the risk of bias assessment are shown in Fig. 2 and Additional file 2: Table S2.

### RBE value

**Fig. 3:**
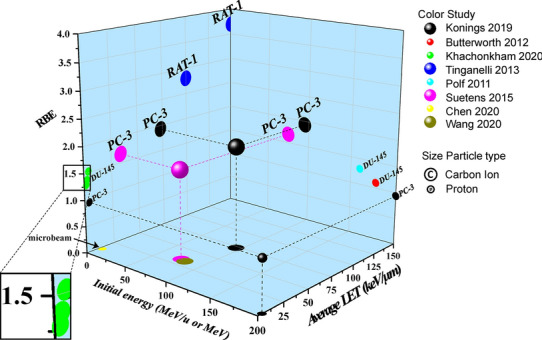
RBE, average LET, initial energy, particle type and cell line of the included studies. *RBE* relative biological effectiveness, *LET* linear energy transfer

RBE value is a parameter that quantitatively expresses the difference in biological effects due to different types of irradiation, which is defined as the dose ratio between the reference photon radiation (usually as 250 kVp X-rays or Co-60 γ-rays) and the particle radiation that produces the same biological endpoint (usually as cell-killing) [37, 38]. In our review, 5 studies declared that they used linear-quadratic model to ascertain RBE values [28, 29, 34–36], the other 7 did not mention what model they used. In total, there were six studies reported 13 RBE values using three kinds of PCa cell lines. Among the 13 RBE values, 9 were proton irradiation (range 0.94–1.52) [26, 32, 34–36], and 4 were carbon ion irradiation (range 1.67–3.7) [28, 29, 36]. Tinganelli et al. reported RBE value for carbon ion irradiation was 2.8 ± 0.2 under normoxia and 3.7 ± 0.1 under anoxia in RAT-1 cell line [29]. Polf et al. reported that the RBE value for proton irradiation alone was 1.3 but could reach to 1.5 when combined with internalized GNPs [35] (Table 2 and Fig. 3).

**Table 2:** RBE values carbon ion/proton in prostate cancer cell lines

Author, year	RBE model	RBE value
Konings 2019	L–Q	PC-3: 0.94 (proton) and 1.93 (carbon ion)
Butterworth 2012	Not reported	DU-145: 1.1
Khachonkham 2020	L–Q	DU-145: 1.28 ± 0.25, 1.37 ± 0.17 and 1.52 ± 0.17 (within SOBP); 1.27 ± 0.27, 0.97 ± 0.4 (before peak)^a^
Tinganelli 2013	L–Q	RAT-1: 2.8 ± 0.2 (oxic) and 3.7 ± 0.1 (anoxic)
Polf 2011	L–Q	DU-145: 1.3 (untreated) and 1.5 (Au-treated)
Suetens 2015	L–Q	PC-3: 1.67

*RBE* relative biological effectiveness, *L–Q* linear–quadratic

^a^ Data were from RBE_10_, RBE_2Gy_ and RBE_4Gy_ were not shown in this table

### Clonogenic Survival

**Fig. 4:**
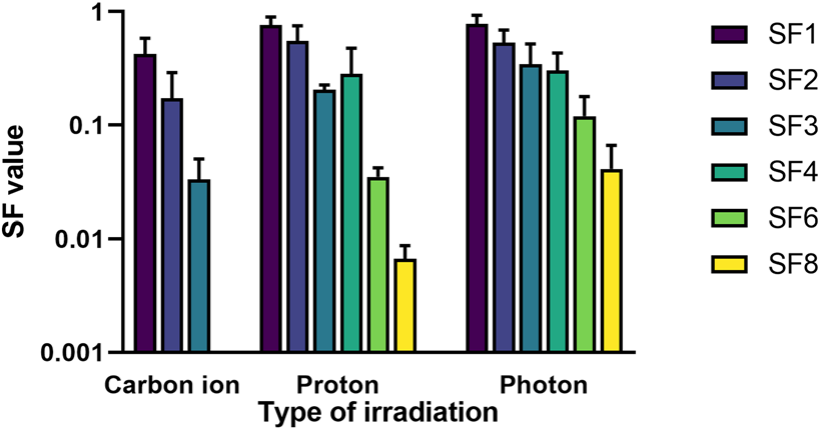
SF values of prostate cancer cells irradiated by carbon ion, proton and photon. *SF* surviving fraction

According to the data given in the studies and prudential calculation conducted by ourselves, we obtained the SF values under different doses of carbon ion, proton, and photon irradiation from ten studies [25, 28–36] (Table 3). For example, SF2 means SF under 2 Gy irradiation. In this review, SF2 were 0.17 ± 0.12, 0.55 ± 0.20 and 0.53 ± 0.16 under carbon ion, proton, and photon irradiation, respectively. Figure 4 clearly indicates that carbon ion irradiation was more effective in clonogenic survival compared with X-rays or protons. The combinations of particle irradiation with drugs such as GANT61, PNKPi and GNPs also enhance the efficacy in cell killing, detailed information will be described in the SER section.

**Table 3:** SF values of prostate cancer cells irradiated by carbon ion, proton and photon

	Carbon ion irradiation	Proton irradiation	Photon irradiation
SF1	0.42 ± 0.16	0.77 ± 0.13	0.78 ± 0.15
SF2	0.17 ± 0.12	0.55 ± 0.20	0.53 ± 0.16
SF3	0.033 ± 0.017	0.21 ± 0.02	0.34 ± 0.18
SF4	0.065 [0.038, 0.092]	0.28 ± 0.19	0.31 ± 0.13
SF5	NA	NA	0.16 [0.01, 0.21]
SF6	NA	0.035 ± 0.007	0.12 ± 0.06
SF8	NA	0.007 ± 0.002	0.041 ± 0.025

*SF* surviving fraction, *NA* not available

Data were mean ± standard deviation or median with interquartile ranges

### The effects of DDR/R

There were 5 studies reported the DDR/R induced by particle irradiation in total [25, 26, 30, 31, 33]. Wang et al. found that carbon ion irradiation induced cell cycle arrest at G0-/G1-phase via overexpression of miR-16-5p [30]. Three studies demonstrated that carbon ion irradiation, alone or combined with drug, can induce cell cycle arrest at G2-/M-phase at different levels [25, 26, 31]. 2 studies investigated the DDR/R in terms of apoptosis. Srivastava et al. proved that combined treatment of carbon ion beam and PNKPi further stimulated apoptosis on the basis of carbon ion irradiation alone through apoptotic body and nucleosomal DNA ladder formation [25]. The other one proved that carbon ion irradiation can lead to higher rates of apoptosis compared to X-ray [31]. Three studies investigated DSB repair. Chen et al. reported relative expression levels of γ-H2A histone family member X (γ-H2AX) were time dependent after proton irradiation [33]. Suetens et al. found dose-dependent increase in γ-H2AX foci numbers and foci occupancy after exposure to carbon ion irradiation [26]. Wang et al. proved that carbon ion irradiation can increase not only γ-H2AX foci numbers but also the foci lasting time and size compared with X-ray [31]. The details are summarized in Table 4.

**Table 4:** DDR/R of prostate cancer cells after carbon ion/proton irradiation

Author, year	Treatment	Outcome	Finds
Wang 2019	Carbon ion irradiation	Cell cycle checkpoints	↑miR‐16‐5p; G0-/G1-phase arrest
Srivastava 2018	Carbon ion irradiation + PNKPi	Cell cycle checkpoints Apoptosis	G2-/M-phase arrest Inducing the apoptosis through apoptotic body and nucleosomal DNA ladder formation
Suetens 2016	Carbon ion irradiation	DSB repair Cell cycle checkpoints	↑γ-H2AX foci numbers and foci occupancy Permanent G2-/M-phase arrest
Chen 2020	Proton irradiation	DSB repair	↑levels of γ-H2AX
Wang 2020	Carbon ion irradiation	DSB repair Cell cycle checkpoints Apoptosis	↑γ-H2AX foci numbers, lasting time and size G2-/M-phase arrest The rates of apoptosis were 27.34% and 37.93% after 2 and 4 Gy carbon ion irradiation, respectively (versus 14.1% and 23.59% following 2 and 4 Gy X-ray irradiation, respectively)

*DDR/R* DNA damage response and repair, *PNKPi* polynucleotide kinase/phosphatase inhibitor, *DSB* DNA double-strand break

↑, increase

### Motility and migration ability

There were only 3 studies investigated the cell motility, migration or their related genes expression after charged particle irradiation. Konings et al. showed carbon ion irradiation displayed a stronger suppression effect regarding migration of PC-3 cells than X-rays and protons by down-regulating *VEGFA* [36]. Suetens et al. reported in two sequential articles that tumor motility-related genes such as *CCDC88A*, *ROCK1*, *NEXN*, *FN1*, *MYH10* and *MYH9* were downregulated after 2 Gy carbon ion irradiation; among them, *CCDC88A*, *ROCK1*, *FN1*, and *MYH9* were also down-regulated after 0.5 Gy carbon ion irradiation [27, 28].

### SER and OER

**Fig. 5:**
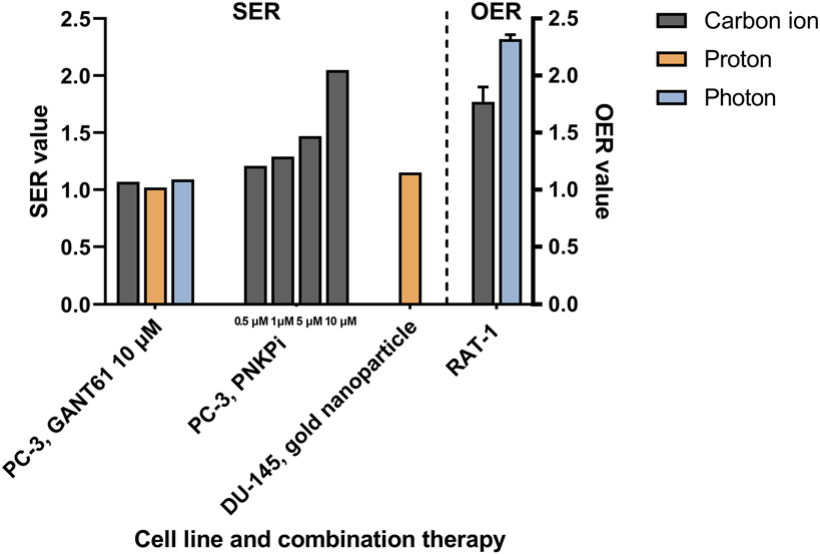
SER values of combination therapy and OER. *SER* standard enhancement ratio, *OER* oxygen enhancement ratio

The SER values of combination therapy were reported in three studies, in which only one study reported the OER. Konings et al. proved that different types of irradiations combined with GANT61 can scarcely enhance the therapeutic effect; the SER values were 1.07, 0.98 and 1.09 for carbon ion, proton and photon irradiation, respectively [36]. Srivastava et al. concluded that the SER values of carbon ion irradiation combined with PNKPi increased with the concentration of the PNKPi in the range of 0.5 to 10 Μm [25]. Polf et al. demonstrated that proton irradiation combined with internalized gold nanoparticles can enhance the efficacy of therapy with a SER value of 1.15 [35]. Tinganelli et al. elaborated that the influence of acute hypoxia and irradiation on RAT-1 cells with a mean OER of 1.77 ± 0.13 for carbon ion and 2.32 ± 0.04 for photon irradiation [29]. The details are presented in Table 5 and Fig. 5.

**Table 5:** SER values of combination therapy and OER

Author, year	Combination therapy (Dose)	Cell line	SER		
			Carbon ion irradiation	Proton irradiation	Photon irradiation
Konings 2019	GANT61 10 μM	PC-3	1.09	0.98	1.07
Srivastava 2018	PNKPi 0.5 μM PNKPi 1.0 μM PNKPi 5 μM PNKPi 10 μM	PC-3 PC-3 PC-3 PC-3	1.21 1.29 1.47 2.05	NA NA NA NA	NA NA NA NA
Polf 2011	Gold nanoparticles	DU-145		1.15	
			OER		
Tinganelli 2013	NA	RAT-1	1.77 ± 0.13	NA	2.32 ± 0.04

*SER* standard enhancement ratio, *OER* oxygen enhancement ratio, *GANT61* GLI antagonist, *PNKPi* polynucleotide kinase/phosphatase inhibitor, *NA* not available

## Discussion

### RBE value

LET is defined as the amount of energy transferred per track. Irradiation with high LET can cause more severe damage to cells, resulting in complicated DNA damage that is difficult to repair. The LET of a particle is influenced mainly by its charge and velocity. In general, more charge and less velocity can lead to a higher LET. That is the reason why carbon ions have a higher LET value than photons and protons. The RBE is an important benchmark that quantifies the difference in biological effects caused by the different LET. A higher RBE means more biological effects at equivalent doses. The most widely reported generic RBE value for protons is 1.1, which is based on a 10% survival rate, mainly used in clinical practice. Here, actually, RBE is a complex function, this value can be determined by various parameters. A growing number of evidences indicate that RBE varies with the LET with respect to given tissue depth, energy, particle type, the dose per fraction, oxygenation state, DNA repair status, cell cycle phase, the biological endpoint and the type of the tissue/cell (α/β ratio) [39, 40]. An in vitro study using the human prostate carcinoma cell line DU-145 irradiated by proton has shown a clear increase in experimental RBE values with LET, especially within the SOBP, with the highest value at the distal edge of the Bragg peak, and a significant decrease for higher doses [34]. The recognized RBE for carbon ion is generally estimated to be 2.5–3, however, values as high as 5 have also been reported [41]. Similarly, the RBE of carbon ion is also determined by these parameters. However, some of these parameters have significant differences in their effects on carbon ion and proton RBE. The RBE of proton with low LET increases slowly with LET value, but the RBE of carbon ion with high LET can reach a maximum at approximately 100–200 keV/μm and then decreases (overkill effect). Furthermore, the RBE of carbon ion is relatively less affected by oxygenation state than that of proton because the OER decreases as the LET increasing [16]. Significantly, the results of a most recent prospective randomized clinical trial revealed that local effect model (LEM) I and α/β = 2 Gy overestimated the RBE of carbon ions in PCa treatment, since RBE-weighted dose was strongly dependent on the α/β ratio as well as the RBE-model [42]. The study concluded that using LEM I with α/β = 4 Gy to adjust the biological dose calculation might be a more practical approach.

### DNA damage and repair

The biological effect was induced by ionization events along the particle track, and these events can cause damage to DNA and other relevant biomolecules. Ionization events caused by photons (low LET irradiation) exhibit both a direct and indirect component. However, high LET-charged particle radiation primarily induced more clustered DNA damage by direct ionizations. The types of clustered DNA damage include chemically altered base lesions (oxidized purines or pyrimidines), abasic sites, intrastrand crosslinks, single-strand breaks (SSBs), and DSBs [43]. Clustered DNA damage is a serious impediment to effective repair mechanisms, with DSBs inside clustered lesions rejoining with slower kinetics and less thoroughly than frank DSBs, resulting in induction of genomic instability [44]. When DNA damage is detected, the corresponding DDR/R mechanism is triggered. The failure of cells to deal with cluster DNA lesions effectively has a significant influence on their normal function and survival. Complex lesions can lead to mutations, the loss of large parts of the genome, and even apoptotic cell death if they are unrepaired or misrepaired [45, 46].

DNA repair pathways in mammalian cells are as follows: base excision repair, nucleotide excision repair, mismatch repair, and the pathways responsible for the repair of DSBs, namely, homologous recombination (HR), classical non-homologous end joining (c-NHEJ), backup non-homologous end joining (b-NHEJ), and single-strand annealing [47]. The selection of the DNA DSB repair pathway is predominantly determined by radiation quality and potentially by DSB load. It is anticipated that the production of DNA lesions with variable degrees of complexity would concurrently activate several DNA repair mechanisms [48, 49]. In low LET irradiation-induced DSB, c-NHEJ is the main pathway in the G1- and early S-phases of the cell cycle, whereas both HR and c-NHEJ were activated in the late S- and G2-phases [50]. It is unclear if cells preferentially select a specific pathway to repair DSBs generated by high LET irradiation [51]. According to some studies, NHEJ is less effective at removing clustered DSBs caused by high LET irradiations than low LET irradiation [52–54]. Saha et al. indicated that the HR pathway may be preferable for the repair of clustered DNA damage caused by heavy charged particles [55]. Gerelchuluun et al. also found that compared to gamma rays and protons, the HR pathway seems to play a more important role in the repair of DSBs in carbon ions [56]. Interestingly, some studies showed that NHEJ may play a main role in carbon ion-induced damage repair, though the using of HR was increased [57–59]. Sridharan et al. indicated that a major complex of c-NHEJ has been implicated in the repair of high-LET radiation-induced clustered DSBs [60]. NHEJ inhibitors have been shown to be more effective than HR after carbon ion irradiation [61, 62]. There were also scholars assumed that NHEJ is essential for processing DNA DSB regardless of the irradiation quality, whereas the significance of HR repair increases when proton irradiation is used [63]. Soni et al. concluded that the choice of pathways may be dose-dependent; when HR becomes saturated under a high dose irradiation, NHEJ could be activated further [64]. This is a critical issue that should be addressed soon as it concerns whether NHEJ inhibitors or HR inhibitors should be used as a combination therapy for CPT.

The genetic background of cell lines may have distinct effects on radiation response for different radiation qualities, genetic defects in DNA repair and DDR genes in cancer cells are important factors [65]. HR-deficient cells and wild-type cells with small interfering RNA-downregulated RAD51 were significantly hypersensitive to proton irradiation, resulting in an elevated relative biological effectiveness compared to the relative biological effectiveness determined for wild-type cells. In contrast, the absence of nonhomologous end-joining did not result in hypersensitivity to proton exposure [66]. Andrea et al. also found human BRCA2-deficient ovarian cancer cells were hypersensitive to proton irradiation compared with photon irradiation [67]. Response to clustered DNA damage repair after particle irradiation is influenced by a transition of ataxia–telangiectasia mutated (ATM) and RAD3-related transition at lesion sites and switch from NHEJ to HR [68–70]. Besides, NHEJ deficiency is more essential than proton LET in determining cell survival. BRCA1 mutation-disrupted cells exhibited increased radiosensitivity for high-LET protons exclusively, whereas RAD51 depletion resulted in increased radiosensitivity for both photons and protons [71].

Histone H2AX, a variation of histone H2A, is one of the key proteins responsible for genome integrity monitoring [72]. H2AX becomes phosphorylated on Ser139 in response to DNA damage, which is defined as γ-H2AX, especially when the damage includes the induction of DSBs [73]. When cluster DNA damage occurs, the marker γ-H2AX can remain for long periods [51]. Ibanez et al. proved that γ-H2AX foci size is an accurate parameter for correlating the rejoining of DSBs induced by different LET radiations and radiosensitivity [74]. The studies included in our review also confirmed that the γ-H2AX level, γ-H2AX foci numbers, occupancy, lasting time and size increased after carbon ion and proton (11.7 keV/µm) irradiation in PC-3 cells [26, 31, 33]. Furthermore, the studies also found that carbon ion irradiation inhibited human PCa cell (LNCaP and PC-3) proliferation by inducing G0-/G1- and G2-/M-phase arrest [25, 26, 30, 31], and eventually resulted in more apoptosis compared to photon irradiation [25, 31].

Epigenetic regulation of DNA repair may also be reliant on radiation quality in addition to proteins directly engaged in DNA DSB rejoining [75–77]. Numerous studies have suggested that ubiquitination, methylation, and acetylation in DNA repair are important epigenetic pathways for targeting in particle therapy [78–82]. A previous study indicated that histone H2B ubiquitylation improves the repair of clustered DNA lesions, resulting in increased survival following exposure to high-LET radiation [79]. Targeting the ubiquitination pattern can thereby sensitize the tumor (high-LET) but not the normal tissue (low-LET) during heavy ion therapy, hence expanding the therapeutic window [77]. The pattern of DNA methylation in cells that survive being exposed to charged particles or X-rays seems to be very different [83]. In addition, histone deacetylase inhibitors appear to promote cell death more efficiently following proton or carbon ion irradiation than following X-ray exposure [81, 82]. All of this evidence seems to indicate that the radiation-induced epigenetic profile is influenced by radiation quality rather than LET alone.

### Motility and migration ability

Although the literatures examining gene expression after exposure to charged particle irradiation on tumor cells are relatively limited, we can still obtain some valuable information about motility, migration or invasion. Fujita et al. found that carbon ion irradiation suppressed the migration and invasion of human pancreatic cells (MIAPaCa-2, BxPC-3 and AsPC-1) via the Rho/ROCK signaling pathway [84]. Akino et al. showed that carbon ion irradiation effectively suppressed migration and invasion of human non-small-cell lung cancer (NSCLC) cells (A549 and EBC-1) via down-regulating *ANLN* [85]. Likewise, proton irradiation has been shown in vitro and in vivo to repress pro-angiogenic gene expression as well as reduce cell motility genes [86, 87]. However, Maruta et al. believed that the motility of A549 cells was increased by carbon ion irradiation via the Rho/ROCK signaling pathway [88]. As mentioned earlier, motility and migration were suppressed in PC-3 cells after carbon ion irradiation by down-regulating several genes, and low expression of *CCDC88A*, *FN1*, *NEXN* and *ROCK1* may be associated with better prognosis [26, 27]. Konings et al. focused on the effects of different radiation types on hedgehog (Hh) signaling pathway and target genes. They concluded carbon ion irradiation suppressed the migration of PC-3 cells more than both photon and proton by down-regulating *VEGFA* [36].

### SER

We also noticed that there were a number of in vitro studies conducted with respect to different perspectives on biological effects of cancer cells induced by charged particles in combination with drugs, including chemotherapy, immunotherapy, nanoparticles and targeted therapy. In targeted therapy combination, many studies investigated a variety of typical targets or signaling pathways in different cancer cells: the combination of poly (ADP-ribose) polymerase (PARP) inhibitors and charged particle irradiation in NSCLC [89, 90], pancreatic cancer [89–91], glioblastoma (GBM) [92], and cervix carcinoma [92] cells; the combination of epidermal growth factor receptor (EGFR) and downstream mammalian target of rapamycin (mTOR) inhibitors and charged particle irradiation in chondrosarcoma [93], hepatocellular carcinoma [94], NSCLC [95], and head and neck squamous cell carcinoma (HNSCC) cells [96]; the combination of heat shock protein 90 (Hsp90) inhibitors and carbon ion irradiation in chondrosarcoma [97], NSCLC [98, 99] and cervix carcinoma [98, 99] cells; and the combination of DNA-dependent protein kinase catalytic subunit (DNA-PKcs) inhibitors and charged particle irradiation in HNSCC [100], breast cancer [57], cervix carcinoma [57], NSCLC [101], and GBM cells [62]. In PCa, Hh inhibitor GANT61 failed to sensitize the cells to proton and carbon ion radiation, with SER values of 0.98 and 1.07; meanwhile, the migration of cancer cells was not inhibited by the combination of two particle irradiation and GANT61 compared with combined with X-ray [36]. Polynucleotide kinase/phosphatase (PNKP) is an enzyme that plays an important role in NHEJ. Srivastava et al. found that when carbon ion irradiation was combined with PNKPi, PC-3 cells experienced considerable apoptosis, and cell cycle arrest was also increased during the G2-/M-phase [25]. Besides, it was proven that metallic-based nano-agents (e.g., NPs, nanocauliflowers, and nanocrystals) expressed radiosensitizing and synergistic effects for radiotherapy. Many studies also demonstrated that nano-agents can be potent in combination with proton [102–107] and carbon ion [108–111] irradiation for the treatment of different malignancies both in vivo and in vitro. Polf et al. showed that the effectiveness of proton radiotherapy for the killing of prostate tumor cells (PC-3) was increased by approximately 15–20% (SER_10_ = 1.15, SER_50_ = 1.2) for those cells containing internalized GNPs [35].

### OER

While there was only one included study that reported the OER of carbon ions, it is still necessary to discuss hypoxia. Hypoxia is very common in malignant solid tumors and is associated with malignancy and a poor prognosis. In radiobiology, oxygen-dependent indirect DNA damage is reduced when hypoxia occurs. A major proportion of this damage is from the production of reactive oxygen species (ROS) (the rest is from the radiolysis of water). When the partial pressure of oxygen (pO2) decreases, fewer oxygen molecules become available, impacting the generation of ROS, which can lead to increased radioresistance. Therefore, the concept of OER was proposed to quantify the radioresistance. The OER of carbon ions decreases with LET at the same oxygen concentration (under 21%) level as well as with oxygen concentration at the same LET value [112, 113]. Hypoxia-inducible factor-1α (HIF-1α; encoded by *HIF1A*) is a transcription factor that regulates several genes in response to hypoxic stimuli, including those involved in tumorigenesis and malignant progression, such as proliferation, metabolic changes, neoangiogenesis, invasion, metastasis, and treatment resistance [114–116]. Several studies demonstrated that carbon ion irradiation reduced HIFs expression in cancer stem cell (CSC) subpopulations of HNSCC cells (SQ20B-CSCs and FaDu-CSCs) and GBM cells (U251, GL15) compared to photon irradiation under hypoxia [117–119]. It was also validated in an in vivo experiment [120]. In general, carbon ion irradiation seems to have promising potential for reducing radioresistance caused by hypoxia, particularly in severely hypoxic malignancies, such as PCa and pancreatic cancer [112].

## Limitations

The findings of this review should be interpreted within the context of its limitations. First, searching only English databases can lead to certain language biases. Furthermore, due to the scarcity of literature reporting radiobiological responses in PCa, as well as the diversification of outcome assessment tools, the validity of our results may be challenged. Lastly, the findings of in vitro studies do not necessarily agree exactly with in vivo studies, more in vivo data accumulation is required. Notwithstanding, we collected the most current information we could obtain, indicating the most recent evidence for charged particle irradiation on PCa in vitro. We hope this review could prompt further fundamental and clinical research regarding this matter.

## Conclusions

To the best of our knowledge, this systematic review is the first study to pool the radiobiological effects of carbon ion and proton irradiation on PCa cell lines, including cell survival (as SF), DDR/R, motility and migration ability, SER and OER. In general, we believe it is plausible to conclude that both carbon ion and proton irradiation have advantages in radiobiological effect over photon irradiation on PCa cell lines. Combination therapy may enhance the gain ratio of CPT for PCa. Based on the information we have right now, carbon ion irradiation seems to have further advantages over proton irradiation.

## Supplementary Information

**Additional file 1**. Specific search strategies for MEDLINE, Embase, and Web of Science.

**Additional file 2**: **Table S1**. Risk of bias scheme. **Table S2**. Results of the risk of bias assessment.

**Additional file 3**. PRISMA 2020 checklist.

## Data Availability

Research data are stored in an institutional repository and will be shared upon reasonable request to the author Tian-Qi Du (dutq21@lzu.edu.cn).

## Abbreviations

PCa: Prostate cancer
RT: Radiation therapy
IMRT: Intensity-modulated radiation therapy
CPT: Charged particle therapy
SOBP: Spread-out Bragg peak
LET: Linear energy transfer
RBE: Relative biological effectiveness
DSBs: Double-strand breaks
OER: Oxygen enhancement ratio
PRISMA: Preferred reporting items for systematic reviews and meta-analyses
DDR/R: DNA damage response and repair
SER: Standard enhancement ratio
SF: Survival fraction
GNPs: Gold (Au) nanoparticles
γ-H2AX: γ-H2A histone family member X
LEM: Local effect model
SSBs: Single-strand breaks
HR: Homologous recombination
NHEJ: Homologous end joining
NSCLC: Non-small-cell lung cancer
GBM: Glioblastoma
HNSCC: Head and neck squamous cell carcinoma
PNKP: Polynucleotide kinase/phosphatase
ROS: Reactive oxygen species
CSC: Cancer stem cell
HIF: Hypoxia-inducible factor
