# Supplementary material for: Does particle radiation have superior radiobiological advantages for prostate cancer cells? A systematic review of in vitro studies
Source: Eur J Med Res. 2022 Dec 26;27:306. doi: 10.1186/s40001-022-00942-2 (PMC9793637; doi:10.1186/s40001-022-00942-2)
Supplement: Supplementary file 1 — Additional file 1. Specific search strategies for MEDLINE, Embase, and Web of Science. [file 40001_2022_942_MOESM1_ESM.docx]

Ovid MEDLINE(R) and Epub Ahead of Print, In-Process, In-Data-Review & Other Non-Indexed Citations, Daily and Versions(R) <1946 to February 18, 2022>

1 exp Heavy Ion Radiotherapy/ 5577

2 exp Proton Therapy/ 4613

3 heavy ion radiotherapy.af. 1260

4 heavy ion therapy.af. 201

5 heavy ion radiation therapy.af. 91

6 particle beam therapy.af. 103

7 carbon ion therapy.af. 333

8 carbon ion radiation therapy.af. 106

9 carbon ion radiotherapy.af. 753

10 carbon ion irradiation.af. 263

11 proton therapy.af. 6885

12 proton radiation.af. 620

13 proton irradiation.af. 767

14 exp Prostatic Neoplasms/ 139899

15 prostatic neoplasms.af. 140444

16 prostate cancer.af. 131538

17 prostatic cancer.af. 6953

18 prostate adenocarcinoma.af. 3083

19 prostatic adenocarcinoma.af. 2889

20 1 or 2 or 3 or 4 or 5 or 6 or 7 or 8 or 9 or 10 or 11 or 12 or 13 9384

21 14 or 15 or 16 or 17 or 18 or 19 175885

22 20 and 21 783

Embase <1974 to 2022 February 18>

1 exp ion therapy/ 2611

2 exp heavy ion radiation/ 791

3 exp heavy ion/ 1609

4 exp proton therapy/ 10489

5 heavy ion therapy.af. 292

6 heavy ion radiotherapy.af. 198

7 heavy ion radiation therapy.af. 104

8 proton therapy.af. 13252

9 proton radiation.af. 5913

10 proton irradiation.af. 1116

11 proton radiotherapy.af. 1059

12 carbon ion therapy.af. 514

13 carbon ion radiation therapy.af. 186

14 carbon ion radiotherapy.af. 1053

15 carbon ion irradiation.af. 351

16 exp prostate cancer/ 240572

17 prostatic neoplasms.af. 10163

18 prostate cancer.af. 258256

19 prostatic cancer.af. 7896

20 prostate adenocarcinoma.af. 13389

21 prostatic adenocarcinoma.af. 3918

22 1 or 2 or 3 or 4 or 5 or 6 or 7 or 8 or 9 or 10 or 11 or 12 or 13 or 14 or 15 21938

23 16 or 17 or 18 or 19 or 20 or 21 280623

24 22 and 23 1637

WOS, BIOSIS, KJD, RSCI, SCIELO Time span = all years, search language = automatic

1. TS=(proton therapy OR proton radiation OR proton irradiation OR proton beam radiation OR proton beam irradiation OR proton radiotherapy)

2. TS=(particle therapy OR heavy ion radiation OR heavy ion irradiation OR heavy ion radiotherapy OR heavy ion therapy OR particle radiation OR particle beam irradiation OR particle radiotherapy OR particle beam radiation)

3. TS=(carbon ion therapy OR carbon ion radiation therapy OR carbon ion irradiation OR carbon ion radiotherapy)

4.TS=(prostatic neoplasms OR prostate cancer OR prostatic cancer OR prostate adenocarcinoma OR prostatic adenocarcinoma)

5. #3 OR #2 OR #1

6. #5 AND #4
