# Supplementary material for: Does particle radiation have superior radiobiological advantages for prostate cancer cells? A systematic review of in vitro studies
Source: Eur J Med Res. 2022 Dec 26;27:306. doi: 10.1186/s40001-022-00942-2 (PMC9793637; doi:10.1186/s40001-022-00942-2)
Supplement: Supplementary file 2 — Additional file 2: Table S1. Risk of bias scheme. Table S2. Results of the risk of bias assessment. [file 40001_2022_942_MOESM2_ESM.docx]

| **Table S1**. Risk of bias scheme. | | | | | |
| --- | --- | --- | --- | --- | --- |
|  |  | **Low** | **Moderate** | **High** | **Unclear** |
| Selection bias | Was the method of cell counting described? | Yes | - | No | - |
| Performance bias | Were the details of irradiation described? | Initial energy, averaged LET, single dose | Described part of them | No | - |
|  | Was the implementation process of experiment reported? | Described every process involved | Described part of them | Not reported | - |
| Detection bias | Were the methods of measuring the results described? | Described every method involved | Described part of them | No | - |
| Attrition bias | Whether the data of experimental results were lost or whether the experiment was not repeated? | No | Lost data or did not repeat | Yes | Not reported |
| Cell related bias | Were the culture conditions of cell reported? | Yes | - | Not reported | - |
|  | Was the cell origin and cell type used reported? | Yes | Cell origin or cell type reported | Not reported | - |
| Other bias | Was there industry sponsoring involved? | No | - | Yes | Not reported |

| **Table S2**. Results of the risk of bias assessment. | | | | | | | | |
| --- | --- | --- | --- | --- | --- | --- | --- | --- |
| **Author (year)** | **Was the method of cell counting described?** | **Were the details of irradiation described?** | **Was the implementation process of experiment reported?** | **Were the methods of measuring the results described?** | **Whether the data of experimental results were lost or whether the experiment was not repeated?** | **Were the culture conditions of cell reported?** | **Were the cell origin and cell type used reported?** | **Was there industry sponsoring involved?** |
| Wang 2019 | High | Moderate | Low | Low | Low | Low | Low | Low |
| Konings 2019 | Low | Low | Low | Low | Low | Low | Low | Low |
| Srivastava 2018 | Low | Low | Low | Low | Low | Low | Low | Low |
| Butterworth 2012 | Low | Moderate | Low | Low | Low | Low | Low | Low |
| Khachonkham 2020 | Low | Low | Low | Low | Low | Low | Low | Low |
| Tinganelli 2013 | High | Moderate | Low | Low | Low | Low | Low | Unclear |
| Suetens 2016 | Low | Low | Low | Low | Unclear | Low | Low | Low |
| Polf 2011 | High | Moderate | Low | Low | Moderate | High | Moderate | Low |
| Suetens 2015 | High | Low | Low | Low | Low | Low | Low | Low |
| Chen 2020 | Low | Low | Low | Low | Unclear | Low | Low | Low |
| Wang 2020 | Low | Low | Low | Low | Low | Low | Low | Low |
| Suetens 2014 | High | Low | Low | Low | Low | Low | Low | Low |
